# T_FR_ Cells Express Functional CCR6 But It Is Dispensable for Their Development and Localization During Splenic Humoral Immune Responses

**DOI:** 10.3389/fimmu.2022.873586

**Published:** 2022-06-22

**Authors:** Cameron R. Bastow, Ervin E. Kara, Timona S. Tyllis, Carola G. Vinuesa, Shaun R. McColl, Iain Comerford

**Affiliations:** ^1^ Chemokine Biology Laboratory, Department of Molecular and Biomedical Science, School of Biological Sciences, The University of Adelaide, Adelaide, SA, Australia; ^2^ Department of Immunology and Infectious Disease, John Curtin School of Medical Research, Australian National University, Canberra, ACT, Australia

**Keywords:** germinal center T cell, T follicular helper (Tfh) cell, CCR6, T follicular regulatory (Tfr) cells, chemokine receptor

## Abstract

Follicular T cells including T follicular helper (T_FH_) and T follicular regulatory (T_FR_) cells are essential in supporting and regulating the quality of antibody responses that develop in the germinal centre (GC). Follicular T cell migration during the propagation of antibody responses is largely attributed to the chemokine receptor CXCR5, however CXCR5 is reportedly redundant in migratory events prior to formation of the GC, and CXCR5-deficient T_FH_ and T_FR_ cells are still capable of localizing to GCs. Here we comprehensively assess chemokine receptor expression by follicular T cells during a model humoral immune response in the spleen. In addition to the known follicular T cell chemokine receptors *Cxcr5* and *Cxcr4*, we show that follicular T cells express high levels of *Ccr6*, *Ccr2* and *Cxcr3* transcripts and we identify functional expression of CCR6 protein by both T_FH_ and T_FR_ cells. Notably, a greater proportion of T_FR_ cells expressed CCR6 compared to T_FH_ cells and gating on CCR6^+^CXCR5^hi^PD-1^hi^ T cells strongly enriched for T_FR_ cells. Examination of *Ccr6^-/-^* mice revealed that CCR6 is not essential for development of the GC response in the spleen, and mixed bone marrow chimera experiments found no evidence for an intrinsic requirement for CCR6 in T_FR_ cell development or localisation during splenic humoral responses. These findings point towards multiple functionally redundant chemotactic signals regulating T cell localisation in the GC.

## Introduction

The GC is a dynamic niche within secondary lymphoid organs that supports immunoglobulin gene mutagenesis in antigen-specific B cells and subsequent selection of B cell clones with increased affinity for antigen. Ultimately, the GC response drives affinity maturation of antigen-specific B cells and generates long-term immunity to pathogens through the formation of memory T- and B-cell subsets and long-lived antibody-secreting plasma cells (LLPCs). Thus, the GC reaction underpins both current vaccination strategies and host protection against invading pathogens, and when perturbed, can result in lymphoma (1) or antibody-mediated autoimmune disease (2). Importantly, the success of the GC rests on interactions between GC B cells (GCB cells) and T follicular helper cells (T_FH_ cells), whilst governance of this process is performed by T follicular regulatory cells (T_FR_ cells) to limit the generation of autoantibodies (3–6). These cellular interactions are highly co-ordinated and governed by dynamic chemotactic signals that underpin the success of the GC response.

T_FH_ cells are a distinct subset of CD4^+^ T cells defined by expression of the master transcription factor BCL6 and chemokine receptor CXCR5 necessary for their localisation in the GC light zone (7–14). Differentiation of T_FH_ cells from naïve CD4^+^ T cell precursors is initiated by dendritic cells in the T cell zone (15, 16) and subsequently reinforced by activated cognate B cells at the T-B border (17, 18). In turn, T_FH_ cells trigger cognate B cell expansion, immunoglobulin class-switch recombination, and direct the differentiation of B cells into an early memory, transient extrafollicular plasmablast (EFPB), or GCB cell lineage (19). Within the GC, GCB cells cycle between a compartmentalized light and dark zone *via* dynamic CXCR4 expression (20, 21), and undergo successive rounds of division and somatic hypermutation targeted within immunoglobulin hypervariable genes to diversify their affinity for antigen (22). Ultimately, competition between arising GCB cell clones for limited T_FH_ help ensures selection of the highest-affinity clones for selection and direction down LLPC and memory B cell differentiation pathways under the instruction of GC T_FH_ cells (23, 24). Critically, the number of T_FH_ cells must be limited to establish a basis for competition and selection of the highest affinity GCB cell clones. This process is regulated in part by T_FR_ cells- a population of follicular T cells that arise from natural T regulatory cells (nTregs) that co-opt the differentiation pathway of T_FH_ cells to gain access to lymphoid niches crucial in the formation of antibody responses (25–27). Given their differentiation from nTregs, T_FR_ cells display oligoclonal specificity for self-antigen and express a suite of immunosuppressive receptors and soluble factors such as neuritin, whilst lacking B-helper molecules (25–29). Together, the T_FR_ cell program lends itself to both restrict T_FH_ and GCB cell expansion prior to GC formation (4, 25–27) and suppress the expansion of non-specific antibodies (3, 4, 30) and autoreactive antibody clones (3–6).

The last two decades have provided great insight to the molecular signaling pathways and migratory events that govern antibody responses. However, the factors facilitating some migratory events and their biological importance remain incompletely understood. Whilst the discovery of CXCR5 expression by T cells with B cell helper function was a was a major step in the identification of *bona fide* T_FH_ cells, CXCR5 expression is only essential for their enrichment in the light zone, not GC entry (14). Whilst down-regulation of CCR7 is essential for T_FH_ cell egress from the T-zone and oxysterol signaling is important in spreading along the T-B border (14, 31, 32), CXCR5-deficient T cells still localize to GCs following immunization (14). Similarly, CXCR5-deficient T_FR_ cells are also capable of localizing to the GC (33). Therefore, additional migratory axes must contribute to the migration of follicular T cell subsets to the appropriate niches throughout the antibody response. Unlike T_FH_ cells, the localisation of T_FR_ cells during each phase of the humoral response is less well characterized. Given that T_FR_ cells display the same differentiation kinetics as T_FH_ cells and also express CXCR5, it has been assumed that T_FR_ cells colocalize with T_FH_ cells throughout the GC response. However, a recent study with human tonsils identified T_FR_ cells were located predominantly outside the GC (34), supporting common observations of scarce Foxp3^+^ cells localized within the GC (25–27, 33, 35). Therefore, the migratory signals that control the localisation of T_FR_ cells remain incompletely understood and we set out to address this gap in knowledge in the current study.

## Methods and Materials

### Mice and Bone Marrow Reconstitutions

C57Bl/6J and Ly5.1 (B6.SJL.*Ptprca*) mice, purchased from the Animal Resource Centre (WA, Australia), and *Ccr6* ^-/-^ mice, previously described (36), were maintained at Laboratory Animal Services, University of Adelaide, under specific pathogen-free conditions. Bone marrow chimeric mice were generated by reconstituting lethally irradiated Ly5.1 mice (1000 Rads) with 5x10^6^ total bone marrow cells from strains described in text. Chimeric mice were immunized following 8 weeks rest. All experiments received approval from the University of Adelaide Animal Ethics Committee.

### Immunization Strategies

Mice were immunized i.p. with 2x10^9^ sheep red blood cells (SRBCs, Applied Biological Products) in endotoxin-free PBS, or 50µg 4-hydroxy-3-nitrophenylacetyl keyhole limpet hemocyanin (NP-KLH)/Alum (Biosearch Technologies), and analyzed at the time points described in text.

### Flow Cytometry and Cell Sorting

Spleens were mashed through 70µm filters and red blood cells were lysed with red blood cell lysis buffer. Splenocytes were counted, stained with LIVE/DEAD dye (Molecular Probes L10119) or Fixable Viability Stain 780 (BD 565388) for dead cell exclusion, then Fc-receptors were blocked with mouse gammaglobulin (Rockland). Surface antibody staining was performed in FACS buffer (PBS/1% BSA/0.04% sodium azide). For CXCR5 staining, splenocytes were incubated with unconjugated anti-CXCR5 (BD 551961), then anti-rat IgG-AF647 (Molecular Probes, A21247) or anti-rat IgG-AF488 (Molecular Probes, A11006), and blocked with rat gammaglobulin (Rockland) before subsequent staining with antibody cocktails. The same process was followed for CCR6 staining with anti-CCR6 (R&D MAB590), and CXCR5 was detected in these samples with biotinylated anti-CXCR5 (BD 551960). The following conjugated antibodies were used: anti-B220 (552771, 562922 and 563103), anti-CD4 (557681, 563106, 563727, 552775, and 557308), anti-CD44 (553133), anti-CD45.2 (560697), anti-CD95 (557653), anti-CD138 (553714 and 558626) and anti-T- and B-cell activation factor (GL7, 562080) from BD; anti-CD3ϵ (25-0031-82), anti-CD38 (17-0381-82), anti-CD44 (12-0441), anti-CD45.2 (45-0454-82), anti-IgD (17-5993-82 and 46-5993-82), anti-Foxp3 (11-5773 and 45-5773), anti-PD-1 (25-9985 and 12-9985), and anti-TCRβ (12-5961) from eBioscience; and anti-B220 (103248), anti-IgD (405725), and anti-neuropillin-1 (145209) from BioLegend. Biotinylated antibodies were detected with streptavidin-BV421 (BD 563259). Intranuclear staining was performed with the Foxp3/Transcription factor staining buffer set (eBioscience). Samples were acquired on a BD LSRII or BD FACSAriaIIIµ, and cell sorting was performed on a BD FACSAriaIIIµ.

### *In Vitro* Chemotaxis

Splenocytes from immunized mice were rested in complete RPMI for 3 hours at 37°C, 5% CO_2_, then harvested and resuspended to 2x10^7^ cells/mL in pre-warmed chemotaxis buffer (RPMI, 0.5% BSA, 25mM HEPES). Synthetic murine CCL20 protein (kindly provided by the late Professor Ian Clark-Lewis) was diluted to concentrations described in text with pre-warmed chemotaxis buffer, then 600μL was loaded into the lower chambers of 24-well HTS Transwell plates (Corning). 100μL of rested splenocytes were loaded into upper permeable supports (5.0μm pore size) and incubated for 3 hours at 37°C, 5% CO_2_. Cells were subsequently harvested for flow cytometric staining and the number of migrated cells was determined with CountBright beads (ThermoFisher). Migration index was calculated from the number of migrated cells for in a given condition/number of migrated cells in no chemokine controls.

### Chemokine Quantitation *via* ELISA

Spleens were mashed in PBS with protease inhibitor cocktail (Roche) and supernatants were stored at -80°C until analysis. CCL20 was quantified from supernatants by ELISA with standard curves using polyclonal rabbit anti-CCL20 generated in-house, as previously described (37).

### Antibody Quantitation *via* ELISA

High-binding EIA/RIA 96-well plates (Costar) were coated overnight with 10µg/mL BSA-NP_32_ or BSA-NP_5_ (Biosearch Technologies Inc) diluted in ELISA coating buffer (28.6mM Na_2_Co_3_, 11.9mM NaHCO_3,_ pH 9.6), or 2µg/mL anti-mouse IgE (BD 553413) diluted in PBS. Wells were subsequently washed four times with 0.05% Tween20/PBS, then blocked with 3% BSA/PBS for 2 hours at room temperature. After washing, sera was serially diluted in 1% BSA/PBS, added to wells and incubated for 2 hours at room temperature. Wells were washed and incubated with anti-mouse IgG-HRP (1030-05), anti-mouse IgM-HRP (1021-05), anti-mouse IgA-HRP (1040-05) from Southern Biotech, or anti-mouse IgE-biotin (BD 553419) diluted in 1% BSA/PBS for 2 hours at room temperature. Biotinylated antibodies were further incubated with streptavidin-HRP (Rockland) for 40 minutes at room temperature. After washing, HRP was detected with 1X TMB ELISA Substrate Solution (eBioscience) and color development was stopped with 1M orthophosphoric acid. Plates were analyzed at 450nm with a Biotrak II plate reader.

### Immunofluorescent Staining

Spleen segments were embedded in O.C.T. compound (Sakura), cut in 12µm thick sections and mounted on polysine coated slides (Thermo Scientific). Sections were fixed in ice-cold 4% PFA for 20 minutes at 4°C, washed, then permeabilized in ice-cold acetone for 10 minutes. After air drying, samples were rehydrated in PBS, blocked with 2% normal mouse and rat serum in staining buffer (PBS with 1% BSA) then stained with anti-CD4 (BD 560468), anti-Foxp3 (eBioscience 13-5773-80) and anti-IgD (eBioscience 17-5993) in staining buffer overnight at 4°C in a humid chamber. Sections were washed and biotin was detected with streptavidin-AF546 (Molecular Probes S-11225). After a final wash, sections were mounted with a coverslip and Vectashield antifade mounting medium (Vector Laboratories), then stored at 4°C in the dark until required for imaging.

### Confocal Microscopy and Image Analysis

Images were acquired on a Leica TCS SP5 confocal microscope with a 20x harmonically corrected plan-apochromat with NA 0.7 water objective using Leica Application Suite: Advance Fluorescence (LAS: AF) software and sequential scanning between frames. Images were processed with FIJI (Image J, National Institutes of Health) for analysis, and Adobe Photoshop CS6 Version 13.0 (Adobe) for presentation, with brightness and contrast adjustments applied equally across images. To enumerate Foxp3^+^ cells, multiple images from the same spleen section were first stitched together in FIJI with the “MosaicJ” plugin. Then regions-of-interest (ROIs) for the periarteriolar lymphatic sheath (PALS) and B cell follicles were manually drawn based on CD4 and IgD staining, respectively. GCs were identified by the lack of IgD staining in areas surrounded by IgD^+^ follicular mantles. The T-B border was defined as the area of the PALS ≤50μm from an adjacent follicle or GC perimeter (17), and this distance was calculated in FIJI with the “Distance Map” function. The T-zone was then defined as the area of the PALS minus the T-B border. Foxp3^+^ cells were identified in FIJI with the “Analyse Particles” function, and larger cell aggregates were manually excluded from analysis. The number of Foxp3^+^ cells in each ROI and the area of each zone were subsequently calculated in FIJI for analyses.

### Statistical Analysis

All data were presented and analyzed in GraphPad Prism 7 with statistical tests performed as described in figure legends. In all cases, p values of <0.05 were considered significant.

### Data Availability

All data from the study are included in the article and/or supporting information.

## Results

### Chemokine Receptor Profiling of Follicular T Cells

**Figure 1:**
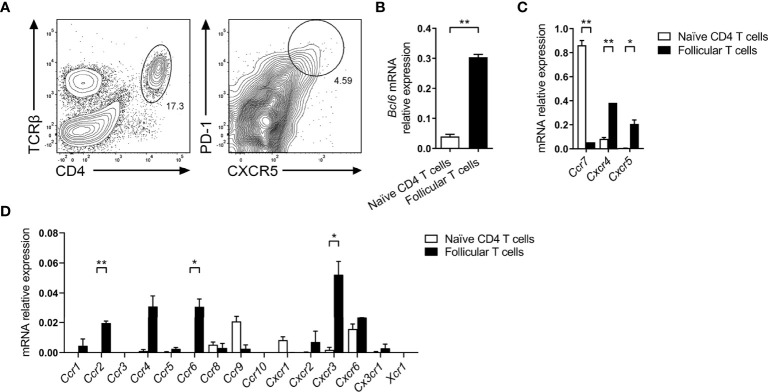
Chemokine receptor gene expression by follicular T cells. **(A)** Representative gating strategy for sorting follicular T cells (CD4^+^TCRβ^+^CXCR5^hi^PD-1^hi^) from day 5 SRBC i.p. immunized mice. Expression of **(B)** follicular T cell master transcription factor *Bcl6*, **(C)** known follicular T cell-tropic chemokine receptors, and **(D)** all other known classical chemokine receptors, between in naïve CD4 T cells (CD4^+^CD44^-^CD62L^+^) and follicular T cells, relative to *Gapdh*. **(B–D)** Data pooled from two independent experiments with 3 mice pooled per time point ± SEM, two-tailed unpaired Student’s t test. *p < 0.05, **p < 0.01.

To comprehensively investigate expression of chemokine receptors by follicular T cells, CXCR5^+^PD-1^+^ T-helper cells were sorted five days post SRBC immunization (**Figure 1A**), and the relative expression of key follicular T cell genes and chemotactic receptors was determined by qPCR. As anticipated, follicular T cells had greater expression of *Bcl6*, encoding the master regulator of follicular T cell differentiation in comparison to sorted splenic naïve CD4 T cells (CD4^+^CD44^-^CD62L^+^; **Figure 1B**). Similarly, follicular T cells had greater expression of transcripts for the follicle/GC-tropic chemokine receptors CXCR5 and CXCR4, and had downregulated *Ccr7* transcript relative to naïve T cells (**Figure 1C**). Having validated the expression of chemokine receptors known to be involved in the follicular T cell program, the relative expression of all other known chemokine receptors was determined. Here, the limited chemokine receptor repertoire of naïve T-helper cells provided a useful biological comparison to evaluate whether follicular T cells had upregulated/downregulated chemokine receptors upon differentiation. Compared to naïve T cells, follicular T cells expressed significantly higher levels of *Ccr2*, *Ccr6*, and *Cxcr3* transcripts (**Figure 1D**). Whilst *Ccr4* appeared upregulated and *Ccr9* downregulated compared to naïve CD4 T cells, these differences were not statistically significant (**Figure 1D**).

### CCR6 is Differentially Expressed by T_FH_ and T_FR_ cells

**Figure 2:**
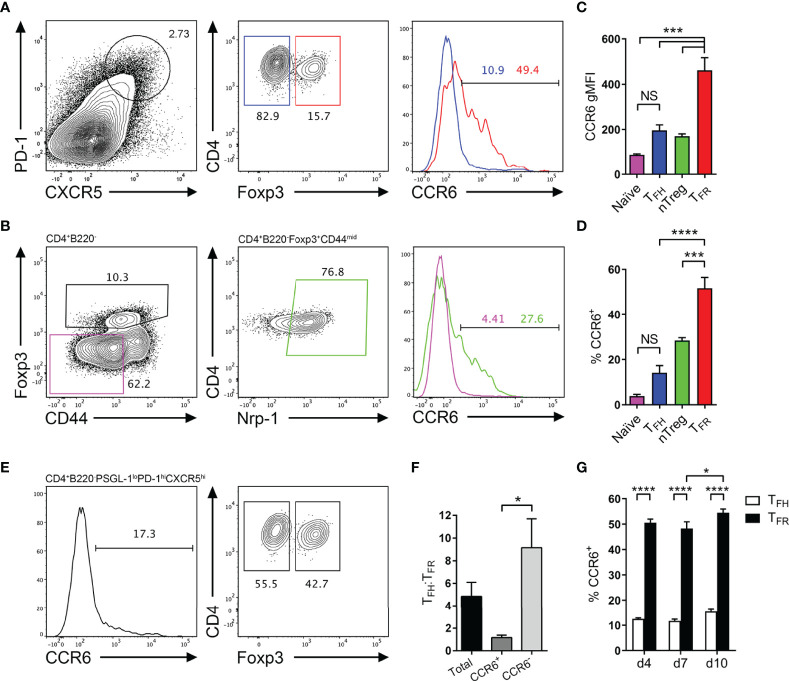
CCR6 expression is highest in T_FR_ cells amongst follicular T cell populations. **(A, B)** Representative gating strategy for CCR6^+^ T_FH_ cells (CD4^+^B220^-^CXCR5^hi^PD-1^hi^Foxp3^-^), T_FR_ cells (CD4^+^B220^-^CXCR5^hi^PD-1^hi^Foxp3^+^), naïve CD4 T cells (CD4^+^B220^-^CD44^lo^Foxp3^-^) and natural T-regulatory cells (nTreg: CD4^+^B220^-^CD44^mid^Foxp3^+^Nrp-1^+^) 6 days after SRBC immunization. **(C)** Geometrical mean fluorescence intensity (gMFI) of CCR6 and, **(D)** percentage of CCR6^+^ cells within populations from **(A)** and **(B)**. **(E)** Representative gating strategy for T_FH_ (Foxp3^-^) and T_FR_ cells (Foxp3^+^) within CCR6^+^ follicular T cells (CD4^+^B220^-^CXCR5^hi^PD-1^hi^CCR6^+^). **(F)** Ratio of T_FH_ : T_FR_ cells within gating strategies from **(A, E)**. **(G)** Frequency of CCR6^+^ T_FH_ and T_FR_ cells at indicated time points after i.p. NP-KLH/Alum immunization. **(A–C, E, F)** Data representative of two independent experiments, n=4 mice ± SEM, one-way ANOVA with Tukey’s multiple comparisons test, **(G)** n=4-5 mice per time-point ± SEM, two-way ANOVA with Sidak’s multiple comparison test. NS, Not significant, *p < 0.05, ***p < 0.001, ****p < 0.0001.

Of the chemokine receptor transcripts significantly upregulated in follicular T cells relative to naïve T cells, an interesting avenue of investigation was determining the role of CCR6 as it is of poorly characterized function with regard to T cell function in the GC and CCR6 expression has been identified in early antigen-engaged B cells (38–41), yet its function in early events underpinning antibody responses is unknown. Furthermore, CCR6 is known to be expressed and utilized by regulatory T cells (42–44), which raised the possibility that T_FR_ cells, which differentiate from nTregs, may express and utilize CCR6. Therefore, the expression and function of CCR6 on T_FH_ and T_FR_ cells was investigated. To interrogate CCR6 expression by follicular T cells, CCR6 was analyzed *via* flow cytometry on T_FH_ cells (CXCR5^hi^PD-1^hi^Foxp3^-^) and T_FR_ cells (CXCR5^hi^PD-1^hi^Foxp3^+^; **Figure 2A**), and respective precursor naïve CD4 T cells and nTregs (**Figure 2B**) following SRBC immunization. Here, nTregs were distinguished from peripheral Tregs by high neuropillin-1 expression (45, 46). Interestingly, CCR6 expression differed significantly between T_FH_ and T_FR_ cell populations by both measures of CCR6 geometric mean fluorescence intensity (gMFI; **Figure 2C**) and proportion of CCR6-positive cells (**Figure 2D**), approximately 15% compared to >50%, respectively. Within precursor populations, few naïve CD4 T cells expressed CCR6, whilst over 25% of nTregs were CCR6-positive (**Figure 2D**), despite the differences in gMFI of CCR6 between these subsets not reaching statistical significance (**Figure 2C**). Collectively, both gMFI and proportion measures of CCR6 expression significantly increased from the precursor nTreg population to effector T_FR_ population (**Figures 2C, D**), potentially implying a role for CCR6 in T_FR_ biology.

Co-expression of additional chemotactic receptors with CXCR5 is known to regulate fine anatomical niche localisation of GC populations during the humoral response (32, 47). Additionally, the ratio of T_FH_ to T_FR_ cells is critical for the correct regulation of antibody responses, and when perturbed, can result in abnormal GC kinetics, affinity maturation and antibody isotype switching, which may drive autoantibody generation (3–6). As different proportions of T_FH_ and T_FR_ cells expressed CCR6, it was determined whether the ratio of T_FH_ to T_FR_ cells was significantly altered when further distinguishing these populations by CCR6 expression. This was investigated by gating CCR6-positive and -negative cells within the follicular T cell gate (CD4^+^B220^-^CXCR5^hi^PD-1^hi^), then delineating T_FH_ and T_FR_ cells by Foxp3 expression (**Figure 2E**). Without any CCR6 pre-gating, the ratio of T_FH_ : T_FR_ cells was approximately 5:1 (**Figure 2F**), as previously reported (26, 35). Interestingly, the T_FH_ : T_FR_ cell ratio significantly differed when segregated by CCR6 expression, from approximately 1:1 within CCR6^+^ populations, to approximately 9:1 within CCR6^-^ populations (**Figure 2F**). This posed a question whether CCR6-driven migratory cues establish finer cellular niches that differentially support or suppress local cell activation through altering the ratio of T_FH_ to T_FR_ cells in that niche. Distinct CCR6 expression patterns between T_FH_ and T_FR_ were not a product of the SRBC immunization strategy, as similar proportions of T_FH_ and T_FR_ cells (15% and 50% respectively) expressed CCR6 following NP-KLH/Alum immunization (**Figure 2G**). Throughout the propagation of the GC response to NP-KLH/Alum, the proportion of CCR6-expressing T_FH_ and T_FR_ cells was steady, with only a slight but significant increase in CCR6-expressing T_FR_ cells from 48% to 54% on days 7 to 10 post immunization (**Figure 2G**). Together, CCR6 protein expression in follicular T cells was confirmed by flow cytometry and differential expression of CCR6 was apparent between T_FH_ and T_FR_ cell subsets.

### CCR6 Expression Facilitates Follicular T Cell Chemotaxis *In Vitro*

**Figure 3:**
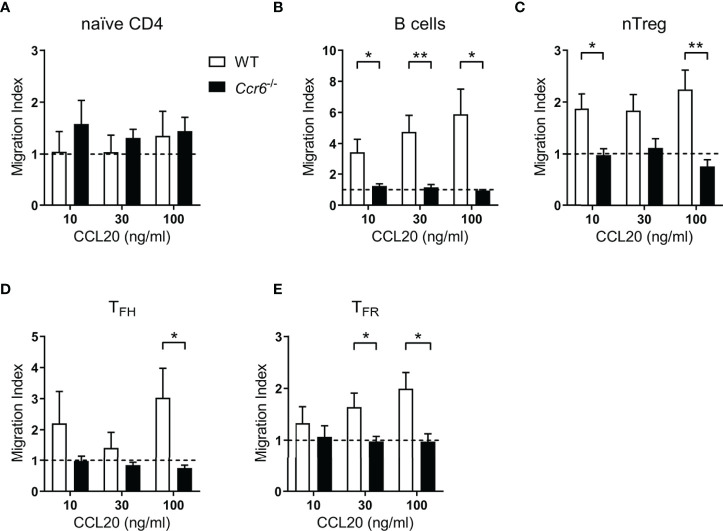
Follicular T cell populations migrate *ex vivo* to CCL20. **(A–E)** Transwell migration of splenocytes from day 6 SRBC immunized WT or *Ccr6* ^-/-^ mice to increasing concentrations of CCL20. Cell populations were identified within total migrated splenocytes by flow cytometry and migration index was calculated relative to controls without chemokine. **(A)** Naïve CD4 T cells: CD4^+^B220^-^CXCR5^-^CD44^lo^Foxp3^-^, **(B)** B cells: CD4^-^B220^+^, **(C)** nTregs: CD4^+^B220^-^CXCR5^-^CD44^mid^Foxp3^+^Nrp-1^+^, **(D)** T_FH_ cells: CD4^+^B220^-^CXCR5^hi^PD-1^hi^CD44^hi^Foxp3^-^, and **(E)** T_FR_ cells: CD4^+^B220^-^CXCR5^hi^PD-1^hi^CD44^hi^Foxp3^+^. Data pooled from two independent experiments, n=3-5 mice/strain ± SEM, two-tailed unpaired Student’s t test between strains at each concentration. *p < 0.05, **p < 0.01.

Given the robust expression of CCR6 by T_FR_ cells, the expression of the sole CCR6 chemokine ligand, CCL20, in the spleen during the response to SRBC immunization was determined. At the peak of the SRBC response, there was an approximately 4-fold increase in extracellular splenic CCL20 protein as determined by ELISA of spleen leach out supernatants (**Supplementary Figure 1A**). Subsequently, it was investigated whether CCR6 expression by T_FH_ and T_FR_ cells facilitated migration to CCL20 *in vitro* using transwell chemotaxis assays of splenocytes from day 6 SRBC-immunized mice. Splenocytes from day 6 SRBC-immunized *Ccr6* ^-/-^ mice were included as a control. Naïve CD4 T cells, which expressed minimal *Ccr6* mRNA (**Figure 1D**) and minimal surface CCR6 protein (**Figures 2B–D**) did not migrate to CCL20 (**Figure 3A**). Conversely B cells, known to express CCR6 and included as a positive-control (41), demonstrated migration to CCL20 in a CCR6- and dose-dependent manner (**Figure 3B**). nTreg cells, approximately a quarter of which express CCR6 (**Figures 2B, D**), demonstrated CCR6-dependent migration towards CCL20 (**Figure 3C**). Both T_FH_ cells (**Figure 3D**) and T_FR_ cells (**Figure 3E**) migrated to CCL20 in a CCR6-dependent manner, demonstrating functional CCR6 expression by these subsets.

### CCR6-Deficient Mice Do Not Display Gross Follicular T Cell Abnormalities or Differences in Splenic Foxp3^+^ Cell Localisation

**Figure 4:**
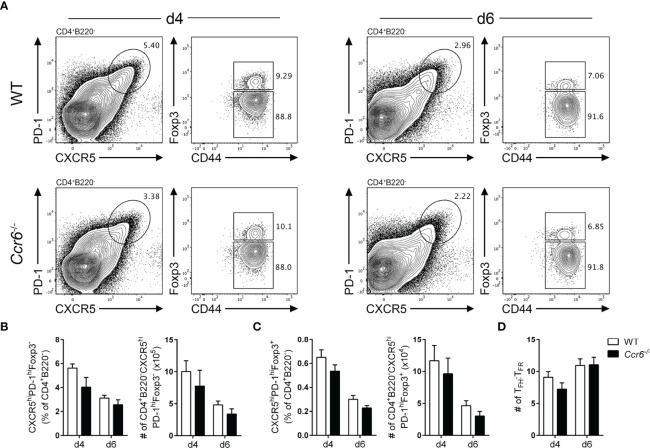
CCR6-deficient mice mount normal T_FH_ and T_FR_ cell responses. **(A)** Representative gating strategy of T_FH_ cells (CD4^+^B220^-^CXCR5^hi^PD-1^hi^CD44^hi^Foxp3^-^) and T_FR_ cells (CD4^+^B220^-^CXCR5^hi^PD-1^hi^CD44^hi^Foxp3^+^) in WT and *Ccr6* ^-/-^ mice four- and six-days post SRBC immunization. Frequency and number of **(B)** T_FH_ and **(C)** T_FR_ cells from **(A)**. **(D)** Ratio of T_FH_ : T_FR_ cells four- and six-days post SRBC immunization in WT and *Ccr6* ^-/-^ mice. **(A–D)** Data representative of 3 independent experiments, n=6-7 mice/time point ± SEM, two-tailed unpaired Student’s t test between strains at each time point.

To investigate the overall contribution of CCR6 on the GC reaction, wildtype and *Ccr6* ^-/-^ mice were immunized with SRBCs and GC populations analyzed at early (day 4) and peak (day 6) time-points. Importantly, CCR6-deficiency does not affect steady-state trafficking of splenic B cells, naïve T cells or Tregs (48–50). At both 4 and 6 days post-immunization there were no significant differences in the proportion or number of naïve CD4 T cells (**Supplementary Figures 2A, B**) or nTregs (**Supplementary Figures 2A, C**) between wildtype and *Ccr6* ^-/-^ mice. There were no significant difference in the proportion or number of T_FH_ (**Figures 4A, B**) and T_FR_ (**Figures 4A, C**) cells between wildtype and *Ccr6* ^-/-^ mice at either time-point analyzed. Consequently, there was no difference between the ratios of T_FH_ : T_FR_ cells between wildtype and *Ccr6* ^-/-^ mice (**Figure 4D**). In line with previous studies (41, 51), CCR6-deficiency had no effect on the number of splenic B cells or primary GCB cells (**Supplementary Figures 3A–C**). However, there was a significant reduction in the proportion of EFPBs during the peak of the response in *Ccr6* ^-/-^ mice (**Supplementary Figure 3D**). Thus, the data indicated that CCR6 was dispensable for the generation of both T_FH_ and T_FR_ cells.

CCR6-deficiency has been previously reported to alter antibody production in the intestinal immune system (52, 53), thus the effect of CCR6-deficiency on splenic antibody production, class switching and affinity maturation was studied following NP-KLH/Alum immunization. Strikingly, *Ccr6* ^-/-^ mice displayed enhanced antibody kinetics as NP-specific IgM titres were significantly elevated in *Ccr6* ^-/-^ mice 7 days post immunization before wildtype IgM titres reached parity on day 14 (**Supplementary Figure 4A**). Similarly, NP-specific IgG titres of both broad-affinity (NP_32_) and high-affinity (NP_5_) were significantly greater in *Ccr6* ^-/-^ mice compared to wildtype mice throughout the response, with a notable 4-fold increase in titres 7 days post-immunization (**Supplementary Figures 4B, C**). Contrary to previous studies (40, 48), affinity maturation was intact in *Ccr6* ^-/-^ mice by measure of NP_5_:NP_32_ ratio, with a small but significant increase in anti-NP IgG affinity during the late stages of the response (**Supplementary Figure 4D**). There were no differences in NP-specific IgA (**Supplementary Figure 4E**), but a trend towards reduced antigen-specific IgE was observed in *Ccr6* ^-/-^ mice 28 days post-immunization (**Supplementary Figure 4F**). Given the importance of T_FR_ cells in restricting the outgrowth of off-target and autoreactive IgE antibodies (29, 30), total IgE titres were measured in immunized wildtype and *Ccr6* ^-/-^ mice. A significant increase in total IgE after immunization was detected in wildtype mice, but not in *Ccr6* ^-/-^ mice (**Supplementary Figure 4G**). Together, these data demonstrated that antibody titres and kinetics in response to NP-KLH were augmented in *Ccr6* ^-/-^ mice, however normal affinity maturation and restricted IgE antibody titres in the absence of CCR6 suggested that T_FR_ cell function did not rely on this chemokine receptor.

**Figure 5:**
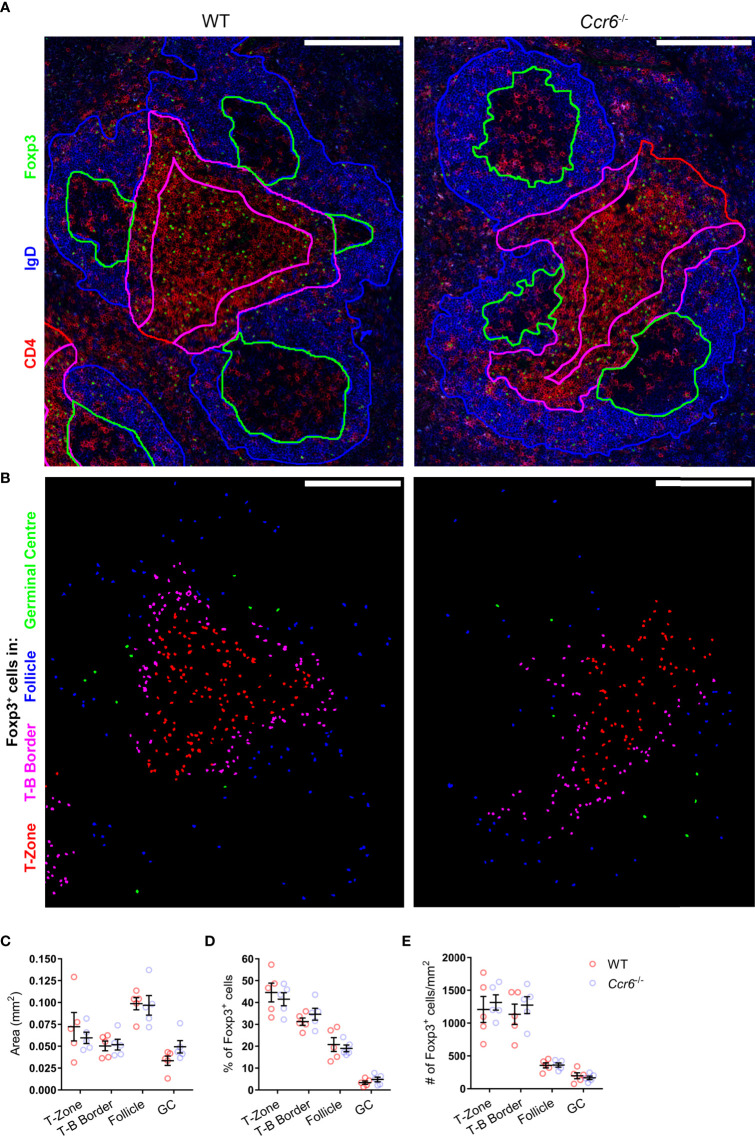
CCR6-deficiency does not affect splenic Foxp3^+^ cell distribution during the peak of SRBC immunization. **(A)** Localisation of Foxp3^+^ cells in PFA and acetone fixed/permeabilized spleen sections from day 6 i.p. SRBC immunized WT and *Ccr6* ^-/-^ mice. Sections were stained with antibodies against CD4 (red), IgD (blue) and Foxp3 (green). Based on CD4 and IgD staining, the following areas are outlined: follicles (blue), GCs (green), T-B border (magenta), and T-zone (red). Scale bar: 200µm. **(B)** Identification of Foxp3^+^ cells from **(A)**, colour-coded based on localisation within the T-zone (red), T-B border (magenta), follicle (blue), or GC (green). **(C)** Average area (mm^2^) of delineated splenic compartments from **(A)**. **(D)** Percentage and **(E)** number/mm^2^ of Foxp3^+^ cells within each splenic niche, quantified from Figure 5.B. **(A, B)** Images representative of n=5 mice/strain, 2-6 images/mouse. **(C–E)** Each dot represents the average of technical replicates per biological replicate, n=5 mice/strain ± SEM. Two-tailed unpaired Student’s t test.

The microanatomical location of T_FR_ cells during the antibody response facilitates interactions with other immune cells and is essential for their regulatory function. Whilst CCR6-deficiency had no effect on the proportion or number of T_FR_ cells, altered positioning of these cells may underpin the heightened antigen-specific antibody responses observed in *Ccr6* ^-/-^ mice. To interrogate the effect of CCR6-deficiency on T_FR_ cell localisation, immunofluorescence microscopy was performed on spleen sections from wildtype and *Ccr6* ^-/-^ mice at the peak of the GC response to SRBC immunization. Utilizing a core stain of antibodies against CD4, IgD and Foxp3, total Foxp3^+^ cells were visualized in spleen sections, and the PALS, T-B border, follicles and GCs were defined (**Figure 5A**). Subsequently, Foxp3^+^ cells residing in each of these areas were identified and enumerated (**Figure 5B**). There was no statistically significant difference in the size (mm^2^) of each compartment between wildtype and *Ccr6* ^-/-^ mice (**Figure 5C**). CCR6-deficiency had no significant effect on Foxp3^+^ cell location within each splenic compartment when quantified as the percentage of total Foxp3^+^ cells in each image (**Figure 5D**), or density (number/mm^2^; **Figure 5E**). Together, the analysis of the GC reactions to SRBC immunization in *Ccr6* ^-/-^ mice revealed no gross defects in T_FH_ or T_FR_ cell biology dependent on CCR6, and suggested that CCR6 deficiency did not affect the microanatomy of splenic niches, nor the gross localisation of splenic Foxp3^+^ cell subsets during a humoral response.

### Cell-Intrinsic CCR6 Expression Is Dispensable for T_FH_ and T_FR_ Differentiation

**Figure 6:**
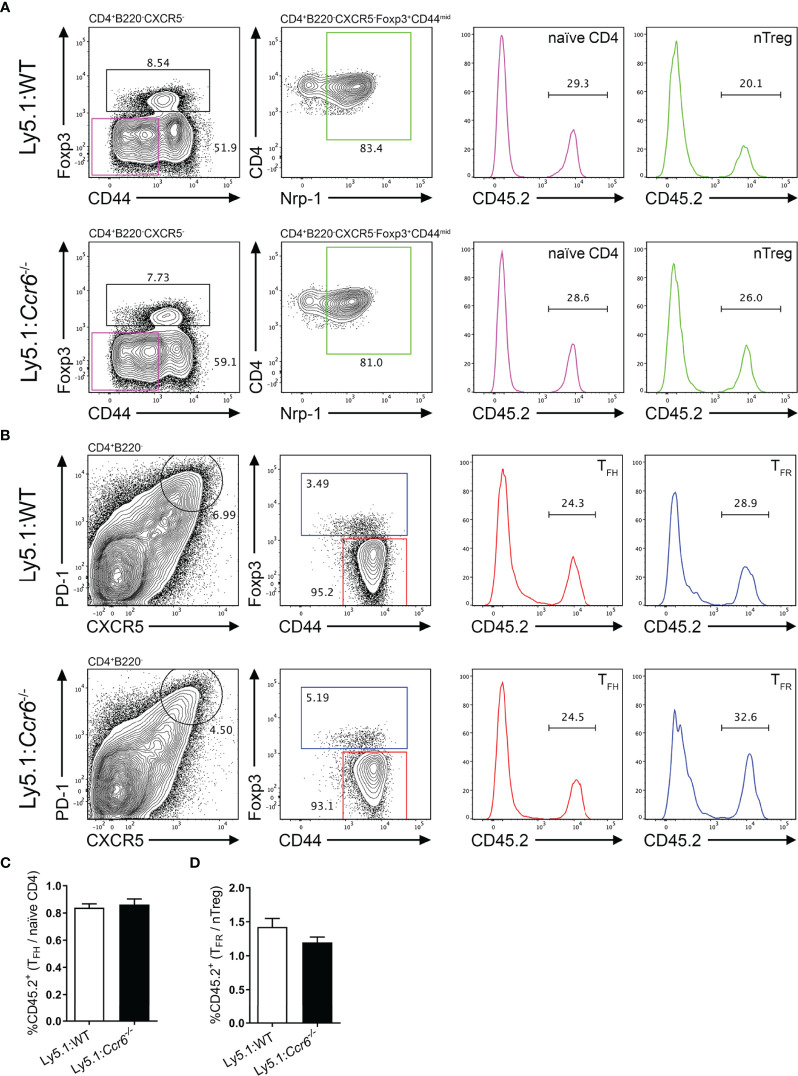
Cell-intrinsic CCR6 function is not required for the formation of T_FH_ and T_FR_ cell populations. Representative gating strategies of **(A)** naïve CD4 T cells and nTregs, and **(B)** T_FH_ and T_FR_ cells from day 6 SRBC immunized irradiated Ly5.1 hosts reconstituted with a 1:1 ratio of Ly5.1:CD45.2^+^ *Ccr6* ^+/+^ (WT, top row) or CD45.2^+^ *Ccr6* ^-/-^ (bottom row) bone marrow. **(C)** Ratio of CD45.2^+^ T_FH_ cells:CD45.2^+^ naïve CD4 T cells in Ly5.1:WT and Ly5.1:*Ccr6* ^-/-^ chimeras. **(D)** Ratio of CD45.2^+^ T_FR_ cells:CD45.2^+^ nTregs in Ly5.1:WT and Ly5.1:*Ccr6* ^-/-^ chimeras. **(A–D)** n=6/chimera group, ± SEM, two-tailed unpaired Student’s t test.

The data described above do not support a role for CCR6 in T_FH_ or T_FR_ cell differentiation and localisation during the GC response. However, extensive cell-cell interactions underpin the differentiation of both follicular T and GCB cell populations. Thus, to eliminate possible CCR6-dependent cell-extrinsic effects that impact on follicular T cell differentiation resultant of global CCR6-deficiency, mixed bone marrow chimeras were generated. Here, irradiated Ly5.1 hosts were reconstituted with 50% Ly5.1 bone marrow (CD45.1^+^), and 50% bone marrow from either WT or *Ccr6* ^-/-^ donor mice (both CD45.2^+^). Resulting chimeric mice contained a WT CD45.1^+^ immune cell compartment present throughout the GC reaction to support the differentiation of CD45.2^+^ WT or CD45.2^+^ *Ccr6* ^-/-^ GC subsets. Therefore, the intrinsic role of CCR6 on the development of GC populations could be determined as knock-on defects resultant of CCR6-deficiency would be eliminated by supporting wildtype CD45.1^+^ cells. Chimeric mice were immunized with SRBC and analyzed by flow cytometry at the peak of the response 6 days later. At this time point, the percentage of CD45.2^+^ naïve CD4 and nTreg precursor populations (**Figure 6A**) were determined in chimeras reconstituted with WT or *Ccr6* ^-/-^ bone marrow. Despite bone marrow reconstitution with 1:1 mix of CD45.1 and CD45.2 bone marrow (WT or *Ccr6* ^-/-^) in irradiated Ly5.1 hosts, there was a disadvantage for CD45.2 bone marrow to differentiate into T cells as <50% of CD4 T cells were CD45.2^+^ (**Figure 6A**). However, as both WT and *Ccr6* ^-/-^ bone marrow were on the CD45.2 genetic background, this disadvantage was controlled for in comparisons between WT and *Ccr6* ^-/-^ GC populations. Thus, the percentage of CD45.2^+^ T_FH_ and T_FR_ cells was determined in WT and *Ccr6* ^-/-^ mixed chimeras (**Figure 6B**) and the ratio of CD45.2^+^ effector population:CD45.2^+^ precursor population was calculated to normalize for any differences in the reconstitution efficiency of CD45.2^+^ bone marrow between individual chimeric mice. As there was no change in the ratio of precursors (naïve or nTreg) to effector cells (T_FH_ or T_FR_ cells) between WT and *Ccr6* ^-/-^ chimeras, this demonstrated that there was no cell intrinsic requirement for CCR6 in the development of T_FH_ cells (**Figure 6C**) or T_FR_ cells (**Figure 6D**). Naïve B cells, GCB cells and EFPBs were also identified in mixed chimeras to discern whether intrinsic CCR6 function regulated GCB cell and EFPB differentiation in primary, polyclonal antibody responses (**Supplementary Figures 5A–D**). There was no selective advantage of CCR6-deficient GCB cells over WT GCB cells (**Supplementary Figure 5B**), however, there was a modest cell-intrinsic involvement of CCR6 in the development of EFPBs (**Supplementary Figure 5D**). Thus, despite its high level of expression, this study could find no role for CCR6 in T_FR_ cell differentiation, function or localisation during T-cell dependent antibody responses in the spleen.

## Discussion

A dominant role for CXCR5 in follicular T cell biology has been well-established but there are likely other chemotactic signals that govern their location. This study identified multiple additional chemokine receptors expressed by follicular T cells. CCR6 was shown to be expressed and functional in follicular T cell subsets but appeared to have a redundant *in vivo* role in the biology of these cells. Whilst CCR6 expression has previously been visualized in follicular T cells (54, 55), here we show substantially increased CCR6 expression by T_FR_ cells compared to T_FH_ cells. Yet, despite robust CCR6 expression in T_FR_ cells, we did not observe any differences in the composition or organization of primary GC reactions within the spleen of CCR6-deficient mice. Previous studies investigating the effect of T_FR_ cell depletion at different timepoints during the GC response have highlighted roles for T_FR_ cells in regulating T_FH_ and GCB cell abundance prior to GC formation, whilst regulation of these populations by T_FR_ cells wanes following the establishment of GCs (4). Thus together, our work suggests that CCR6 may not play a role facilitating organization or interactions between T- and B-cell populations to regulate the early stages of the immune response. This is supported by our observation that CCR6-deficiency does not imbue an intrinsic advantage to T_FH_ cells or GCB cells in mixed chimeras. These data are in line with previous studies investigating the role of B cell CCR6 expression in primary immune responses where no differences in GCB cell proportions were observed between WT and *Ccr6* ^-/-^ mice (41, 51), nor between WT and CCR6-deficient transgenic hen egg lysozyme-specific B cells (56).

In this study, CCR6-deficiency resulted in a significant increase in the kinetics and titres of antigen-specific IgM and IgG. Increased antigen-specific IgM titres in *Ccr6* ^-/-^ mice have previously been described following subcutaneous KLH immunisation (53), however our data here demonstrate the transient nature of this increase. Similarly, *Ccr6* ^-/-^ mice displayed rapid and robust production of IgG early in the antibody response against NP-KLH/Alum and significantly greater titres were maintained throughout the GC response with affinity maturation remaining intact. Despite these results, the effect of CCR6-deficiency on IgG responses remains unclear. Previous studies have identified increased IgG1 titres in CCR6-deficient mice at the expense of affinity maturation (40, 48), whilst others have identified no differences in the primary antibody response (41, 51) or defects in IgG3 responses (53). As class switch recombination occurs prior to activated B cell fate trifurcation at the T:B border (57), the cellular source of increased antigen-specific IgG observed here remains an open question. Future work should consolidate immunization strategies to identify whether B cell CCR6 activity functions in a context-specific setting to enhance antibody kinetics, titres, isotype switching and affinity maturation. In line with previous allergy models in the eye and lungs (58–60), CCR6-deficiency impaired total IgE responses upon intraperitoneal NP-KLH/Alum immunization. In models of pulmonary allergy, T-cell CCR6 function was implicated in the production of IgE (60). Similarly, a population of CCR6^+^IL-10^+^ memory T cells distinct from regulatory and follicular T cell subsets was recently identified with the ability to trigger IgE switching in B-cells (61). As we also observe impaired IgE titres in *Ccr6* ^-/-^ mice, collectively these studies implicate the CCR6-CCL20 axis in necessary differentiation and/or survival signals for IgE-switched antibody secreting cells. Furthermore, given the described roles of T_FR_ cells in supporting the production of antigen-specific IgE and suppressing the production of non-specific IgE clones (29, 30), CCR6^+^ T_FR_ cells may function within such a niche to regulate the balance of antigen-specific:off-target IgE antibody clones. Together our data highlights a role for CCR6^+^ cells in the induction of IgE and warrants further investigation into the precise identity and localisation of CCR6+ cells during antibody responses and IgE-mediated pathologies.

One caveat to the present study is that a relatively modest induction of CCL20 was observed in the spleen following the immunization strategies utilized. It is possible that a role for CCR6 may be apparent in other scenarios that more strongly induce CCL20 expression in secondary lymphoid tissue. Other inflammatory models could potentially be utilized which have been described to induce more pronounced splenic CCL20 induction. Notably, robust CCL20 induction has been identified in the spleen following immunization with synthetic peptidoglycan compounds (48). In this setting, CCL20 expression was induced in radio-resistant cells downstream of TNFα signaling, which has been demonstrated to induce CCL20 expression in a variety of different cell types (62–64). The immunization strategies utilized in this study, SRBC and NP-KLH/Alum, may not sufficiently induce splenic CCL20 expression to reveal CCR6-dependent migratory events as alum does not induce nor act through TNFα (65, 66), and SRBC immunization initiates antibody responses directly through missing-self CD47 and SIRPα interactions between xenogeneic red blood cells and splenic dendritic cells, respectively (67). Whether splenic follicular T cell CCR6 function would be apparent in more inflammatory settings such as bacterial infection remains an open avenue for investigation.

Another possibility is that CCR6 function on follicular T cells is required in different secondary lymphoid microenvironments not present in the spleen. Steady-state CCL20 expression has been identified in the sub-epithelial dome (SED) of Peyer’s patches (53, 68, 69), and is further upregulated by ingested bacterial products from *Salmonella* species and *Listeria monocytogenes (* 70). Here, CCR6 mediates the migration of B cells and dendritic cells in to the SED and facilitates crucial interactions between these cells necessary for class-switch recombination (52), exemplified by diminished IgA responses to gut microbes in *Ccr6* ^-/-^ mice (53). In the Peyer’s patches, T_FR_ cells are crucial in diversifying IgA against gut microbiota and establishing a regulatory loop whereby the healthy microbiome established by T_FR_ cells supports Foxp3^+^ cells and IgA production (71). Therefore, it is conceivable that T_FR_ cells may also utilize CCR6 to migrate to the SED and influence the diversification of IgA antibodies and microbiota through interactions with dendritic cells and B cells in this niche.

Steady-state CCL20 expression has also been identified in the lymph node subcapsular sinus (SCS) (72, 73) and is further upregulated following SIV infection (73) or LPS administration (74). At the SCS, CCL20 attracts innate-like lymphocytes (72), and it is hypothesized that high CCR6 expression by memory B cells may contribute to their peri-subcapsular localisation in lymph nodes during the steady-state (75). Upon secondary antigen exposure, memory B cells in the peri-subcapsular space interact with memory T_FH_ cells, proliferate, and differentiate into plasma cells (32, 75). CCR6-deficiency results in diminished memory B cell responses characterized by reduced plasma cell differentiation and antibody titres upon secondary challenge (48, 51). Additionally, early-activated B cells upregulate CCR6 and accumulate at the SCS/follicle interface to proliferate during the early phases of the immune response prior to the formation of the GC (17). The importance of localisation adjacent to the SCS for rapid B cell expansion prior to seeding the GC is incompletely understood, as is early CCR6 upregulation by antigen-activated B cells. However, B cells that accumulate at the SCS do not express Bcl6 and upregulate intracellular immunoglobulin light chain expression, consistent with EFPB differentiation (17). Together, given that T_FR_ cells restrict the emergence of autoantibodies following immune challenge (3–6), CCR6 may facilitate the recruitment of T_FR_ cells to subcapsular niches to regulate crucial proliferation and differentiation events that underpin plasma cell differentiation. As the lymph node subcapsular niche is utilized by both early-activated and memory B cells, T_FR_ cells may colocalize to this niche to regulate both primary and memory antibody responses and/or limit autoantibody emergence. This may explain why T_FR_ cells in human tonsils were predominantly identified outside of the GC (34).

An open question remains as to potential roles of other chemokine receptors in contributing to follicular T cell homing in addition to CXCR5. This study revealed higher expression of *Ccr2* and *Cxcr3* relative to naïve CD4 T cells, and there was a strong trend towards greater expression of *Ccr4* in follicular T cells. Indeed, a recent study by Liu et al. identified CCR4 expression by T_FH_ and T_FR_ cells was necessary to facilitate interactions between high-affinity GCB cells and GC T_FH_ cells (76). A recent study also identified *Ccr2* expression in neonatal GC T cells (77), however its function in GC biology remains unknown. In Tregs, CCR2 has been demonstrated to regulate CD25 expression and thus sensitivity to IL-2 signalling (78). Given that IL-2 signaling is detrimental to the stability of the follicular T cell transcriptome (3, 79, 80) and T_FR_ cells downregulate CD25 (3, 79), CCR2 expression in follicular T cells may further fine-tune IL-2 signaling. Multiple studies have identified CXCR3 expression by T_FH_ cells during antibody responses to viral infection (81–83). Conversely, scarce CXCR3 expression by T_FR_ cells was identified on during initial characterization of this subset (25). CXCR3 ligand CXCL9 is induced in the interfollicular/outer-follicle areas of the lymph node and spleen upon inflammation (84), both of which are key niches in the early development of humoral responses (17, 85). Indeed, dysregulated CXCR3 expression in T_FH_ cells relocated GC T_FH_ cells into the follicle (47). Whether CXCR3 expression by T_FH_ cells plays key roles during the initiation of GCs remains an open avenue of investigation. Together, these observations justify future investigation into the role of additional chemokine axes in follicular T cell biology.

## Data Availability Statement

The original contributions presented in the study are included in the article/**Supplementary Material**, further inquiries can be directed to the corresponding author/s.

## Ethics Statement

The animal study was reviewed and approved by University of Adelaide Animal Ethics Committee.

## Author Contributions

CRB designed, performed and analyzed experiments, and wrote the manuscript. EEK designed, performed and analyzed experiments. TST performed experiments. CGV provided expertise and helpful discussions. SRM and IC designed experiments, supervised the study and wrote the manuscript. All authors contributed to the article and approved the submitted version.

## Funding

This study was supported by NHMRC project grant 1163335. CB was supported by an Australian Postgraduate Award. IC is supported by a senior research fellowship from MS Australia.

## Conflict of Interest

The authors declare that the research was conducted in the absence of any commercial or financial relationships that could be construed as a potential conflict of interest.

## Publisher’s Note

All claims expressed in this article are solely those of the authors and do not necessarily represent those of their affiliated organizations, or those of the publisher, the editors and the reviewers. Any product that may be evaluated in this article, or claim that may be made by its manufacturer, is not guaranteed or endorsed by the publisher.
