# Supplementary material for: TFR Cells Express Functional CCR6 But It Is Dispensable for Their Development and Localization During Splenic Humoral Immune Responses
Source: Front Immunol. 2022 Jun 22;13:873586. doi: 10.3389/fimmu.2022.873586 (PMC9257258; doi:10.3389/fimmu.2022.873586)
Supplement: Supplementary file 1 [file DataSheet_1.pdf]

frontiers

Supplementary Material

T_{FR} cells express functional CCR6 but it is dispensable for their development and localization during splenic humoral immune responses

Cameron R. Bastow, Ervin E. Kara, Timona S. Tyllis, Carola G. Vinuesa, Shaun R. McColl, Iain Comerford

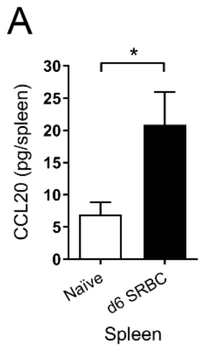

Supplementary Figure 1: Splenic CCL20 is increased following SRBC immunization

(A) Quantity of CCL20 (pg) in spleen supernatant from steady-state and day 6 SRBC-immunized WT mice, as determined by ELISA. Data pooled from two independent experiments, n=6/condition ±SEM, two-tailed unpaired Student's t test with Welch's correction. *p<0.05.

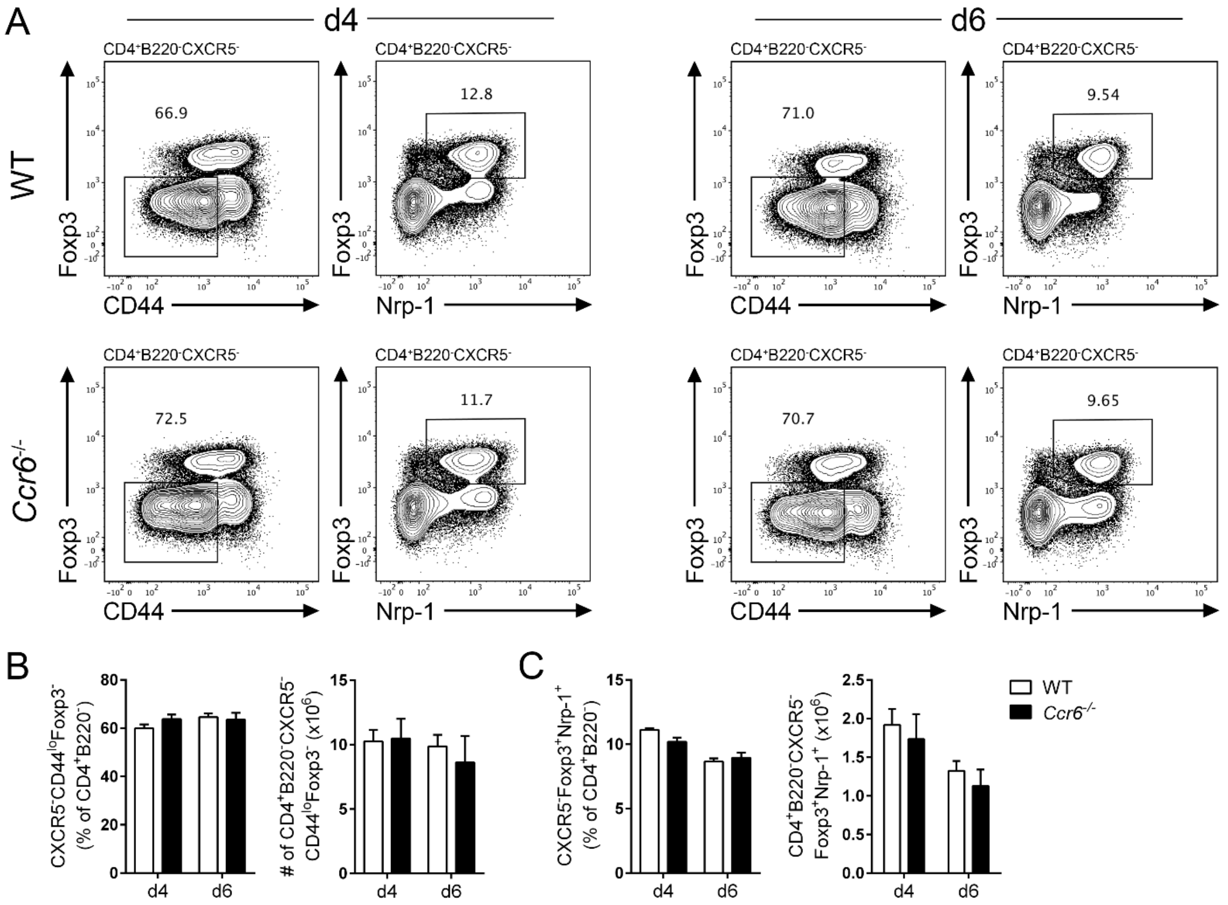

Supplementary Figure 2: Splenic naïve CD4 T cell and Treg populations are unaltered in *Ccr6*$^{-/-}$ mice following SRBC immunization.

(A) Representative gating strategy of naïve CD4 T cells ($CD4^{+}B220^{-}CXCR5^{-}CD44^{lo}Foxp3^{-}$) and nTregs ($CD4^{+}B220^{-}CXCR5^{-}Foxp3^{+}Nrp\text{-}1^{+}$) four and six days post SRBC immunization in WT and *Ccr6*$^{-/-}$ mice. Frequency and number of naïve CD4 T cells **(B)** and nTregs **(C)** from (A). (A-C) Data representative of 3 independent experiments, n=6-7 mice/time point ±SEM, two-tailed unpaired Student's t test between strains at each time point.

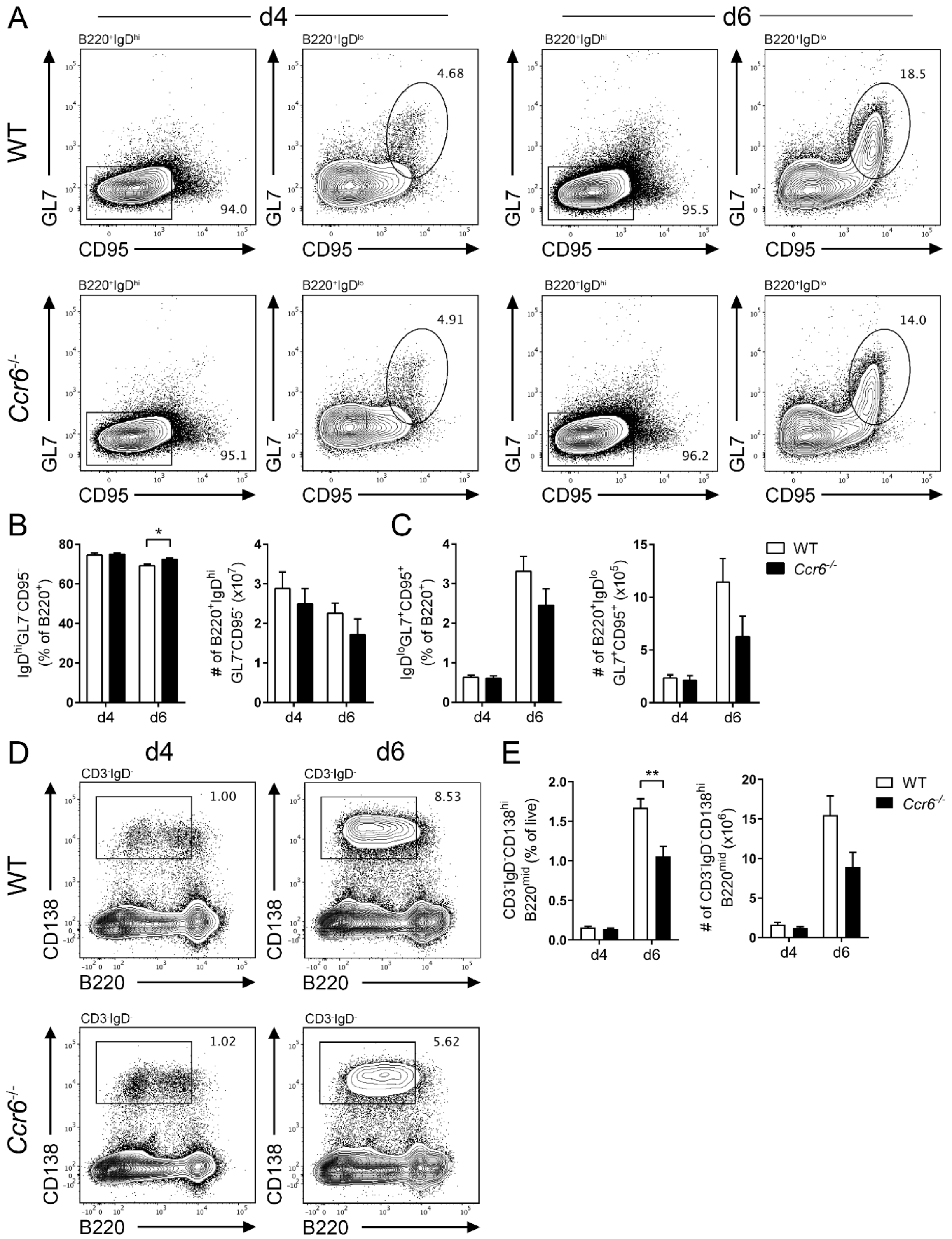

Supplementary Figure 3: Splenic EFPB responses are altered in *Ccr6*$^{-/-}$ mice following SRBC immunization.

(A) Representative gating strategy of naïve B cells (B220^{+}IgD^{hi}GL7^{-}CD95^{-}) and GCB cells (B220^{+}IgD^{lo}GL7^{+}CD95^{+}) 4 and 6 days post SRBC immunization in WT and *Ccr6*$^{-/-}$ mice. Frequency

and number of **(B)** naïve B cells, and **(C)** GCB cells from (A). **(D)** Representative gating strategy of EFPBs ($CD3^{-}IgD^{-}CD138^{hi}B220^{mid}$) 4 and 6 days post SRBC immunization in WT and *Ccr6*$^{-/-}$ mice. **(E)** Frequency and number of EFPBs from (D). (A-E) Data representative of 3 independent experiments, n=4-7 mice/time point ±SEM, two-tailed unpaired Student's t test between strains at each time point. *p<0.05, **p<0.01.

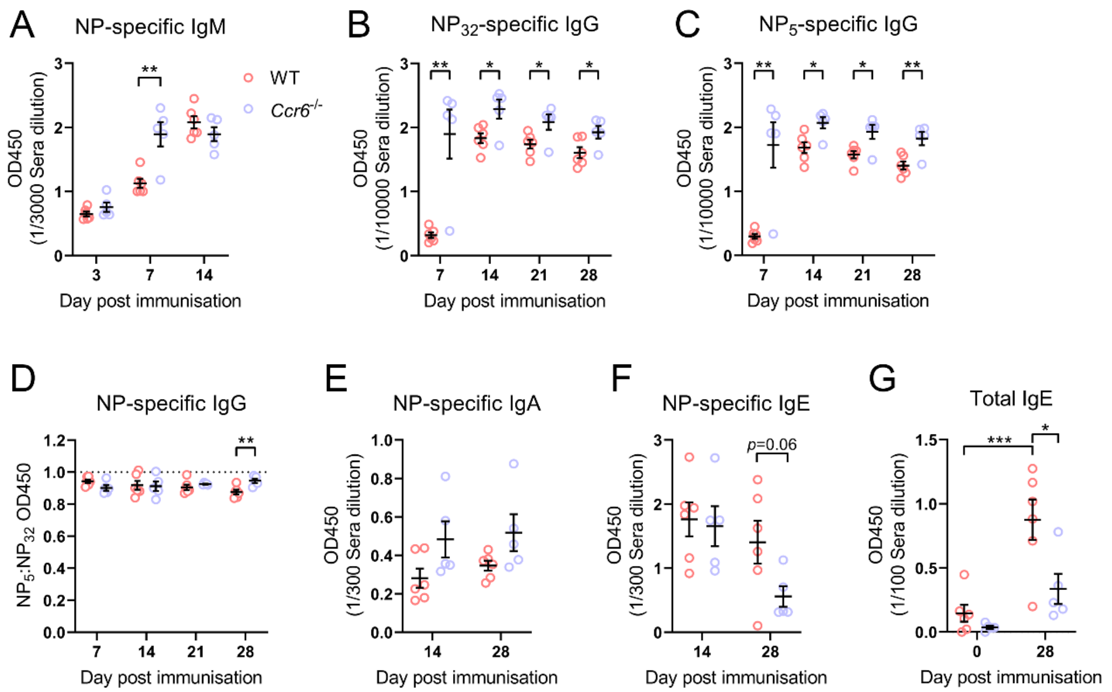

Supplementary Figure 4: *Ccr6*$^{-/-}$ mice display enhanced antigen-specific antibody kinetics and titres following NP-KLH/Alum immunization.

(A) NP-specific IgM antibody levels in the sera of wildtype and *Ccr6*$^{-/-}$ mice at indicated time points following NP-KLH/Alum immunization. NP-specific IgG antibodies with **(B)** broad-affinity (NP_{32}) and **(C)** high-affinity (NP_5) in the sera of wildtype and *Ccr6*$^{-/-}$ mice at indicated time points following NP-KLH/Alum immunization. **(D)** Affinity maturation of NP-specific IgG antibodies in wildtype and *Ccr6*$^{-/-}$ mice during the GC response as measured by the ratio of NP_5:NP_{32} NP-specific antibodies. NP-specific **(E)** IgA and **(F)** IgE in the sera of wildtype and *Ccr6*$^{-/-}$ mice at the peak and late stages of the antibody response to NP-KLH/Alum. **(G)** Total IgE in the sera of unimmunized and NP-KLH/Alum immunized wildtype and *Ccr6*$^{-/-}$ mice. (A-G) n=5-6 mice/timepoint ±SEM, (A-F) two-tailed unpaired Student's t test between strains at each time point, (G) two-way ANOVA with Sidak's multiple comparison test. *$p<0.05$, **$p<0.01$, ***$p<0.001$.

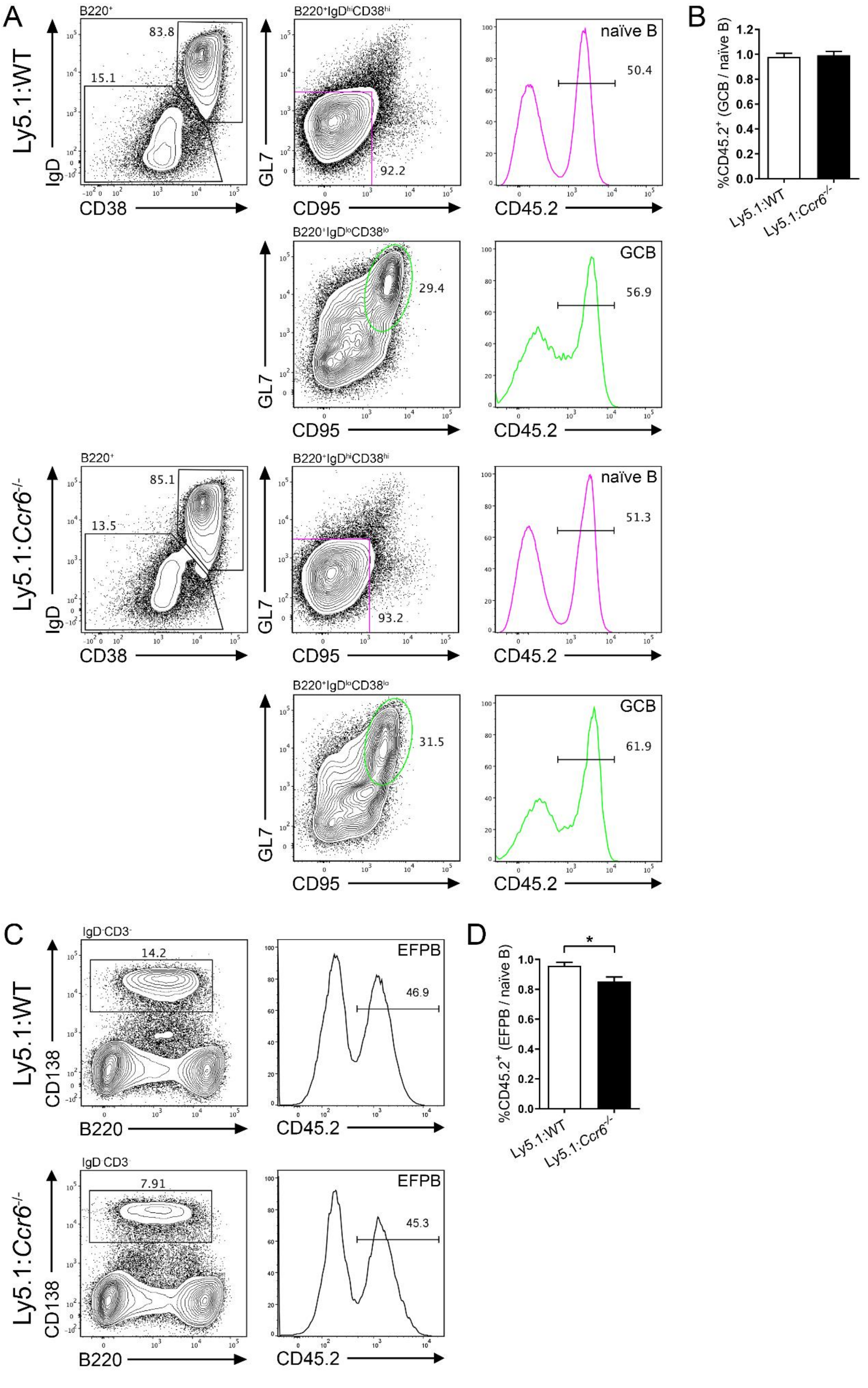
A
B
C
D
Ly5.1:WT
Ly5.1:Ccr6-/-
B220+
IgD
CD38
83.8
15.1
85.1
13.5
B220+IgDhiCD38hi
B220+IgDloCD38lo
GL7
CD95
92.2
93.2
29.4
31.5
CD45.2
naïve B
GCB
50.4
56.9
51.3
61.9
%CD45.2+ (GCB / naïve B)
IgD-CD3-
CD138
B220
14.2
7.91
EFPB
46.9
45.3
%CD45.2+ (EFPB / naïve B)
*

Supplementary Figure 5: Cell-intrinsic CCR6 function is required for optimal EFPB differentiation

(A) Representative gating strategies for CD45.2$^+$ naïve B cells and GCB cells 6 days post SRBC immunization in irradiated Ly5.1 hosts reconstituted with a 1:1 ratio of Ly5.1:WT (top row) or Ly5.1:*Ccr6*$^{-/-}$ (bottom row) bone marrow. **(B)** Ratio of CD45.2$^+$ GCB cells:CD45.2$^+$ naïve B cells in Ly5.1:WT and Ly5.1:*Ccr6*$^{-/-}$ chimeras. **(C)** Representative gating strategies for CD45.2$^+$ EFPBs 6 days post SRBC immunization in irradiated Ly5.1 hosts reconstituted with a 1:1 ratio of Ly5.1:WT (top row) or Ly5.1:*Ccr6*$^{-/-}$ (bottom row) bone marrow. **(D)** Ratio of CD45.2$^+$ EFPBs:CD45.2$^+$ naïve B cells in Ly5.1:WT and Ly5.1:*Ccr6*$^{-/-}$ chimeras. (A,D) n=6/chimera group, ±SEM, two-tailed unpaired Student's t test. *p<0.05.
